# Characterisation and antifungal activity of extracellular chitinase from a biocontrol fungus, *Trichoderma asperellum* PQ34

**DOI:** 10.1080/21501203.2019.1703839

**Published:** 2019-12-14

**Authors:** Nguyen Hoang Loc, Nguyen Duc Huy, Hoang Tan Quang, Tran Thuy Lan, Tran Thi Thu Ha

**Affiliations:** a Institute of Bioactive Compounds and Department of Biotechnology, University of Sciences, Hue University, Hue, Vietnam; b Department of Applied Biology and Biotechnology, Institute of Biotechnology, Hue University, Hue, Vietnam; c Department of Plant Protection, University of Agriculture and Forestry, Hue University, Hue, Vietnam

**Keywords:** Antifungal activity, chitinase, *Colletotrichum* sp, *Sclerotium rolfsii*, *Trichoderma asperellum*

## Abstract

*Trichoderma* species were known as biological control agents against phytopathogenic fungi because they produce a variety of chitinases. Chitinases are hydrolytic enzymes that break down glycosidic bonds in chitin, a major component of the cell walls of fungi. The present study shows that extracellular chitinase activity reached a maximum value of approximately 22 U/mL after 96 h of *T. asperellum* PQ34 strain culture. The optimal temperature and pH of enzyme are 40°C and 7, respectively, whereas the thermal and pH stability range from 25°C to 50°C and 4 to 10, respectively. Chitinase at 60 U/mL inhibited nearly completely *in vitro* growth of *Colletotrichum* sp. (about 95%) and *Sclerotium rolfsii* (about 97%). In peanut plants, 20 U/mL of chitinase significantly reduced the incidence of *S. rolfsii* infection compared to controls. The fungal infection incidence of seeds before germination and 30 days after germination was only 2.22% and 2.38%, while the control was 13.33% and 17.95%. Besides, chitinase from *T. asperellum* PQ34 can also prevent anthracnose that is caused by *Colletotrichum* sp. on both mango and chilli fruits up to 72 h after enzyme pre-treatment at 40 U/mL. In mango and chilli fruits infected with anthracnose, 40 U/mL dose of chitinase inhibited the growth of fungi after 96 h of treatment, the diameter of lesion was only 0.88 cm for mango and 1.45 cm for chilli, while the control was 1.67 cm and 2.85 cm, respectively.

## 1. Introduction

Genus *Trichoderma* (Hyphomycetes) is widely distributed in nature, especially in soil (Harman et al. 2004) and endophyte (Baiyee et al. 2019a). They have been used in the production of enzymes and biological controls of plant diseases (Samuels 1996). *Trichoderma* species excrete enzymes such as chitinases, β-glucanases and proteases; these enzymes are induced during interactions between *Trichoderma* and cell walls of phytopathogenic fungi (Wonglom et al. 2019; Baiyee et al. 2019b). Although other enzymes may be also involved in the complete degradation of cell walls of phytopathogenic fungi, chitinase is generally considered an important enzyme since its substrate, chitin, is the most abundant component in cell walls of many fungus species (Baek et al. 1999). Chitinases (E.C 3.2.2.14) are glycosyl hydrolases with the molecular weight ranging from 20 to 90 kDa. They are present in a wide range of organisms such as bacteria, fungi, yeasts, plants, actinomycetes, and arthropods (Hamid et al. 2013). Chitinases can degrade chitin into low molecular weight oligosaccharides (Kramer and Muthukrishnan 1997). Chitin (poly 1,4-β-N-acetylglucosamine) is the second most abundant polysaccharide (after cellulose) occurring in nature and is a long-chain polymer of N-acetylglucosamine (2-acetamido-2-deoxy-D-glucose) linked by β-1,4 bonds (Song et al. 2005). Chitin is the building material that gives strength to the exoskeletons of crustaceans, insects, and the cell wall of fungi (Elieh-Ali-Komi and Hamblin 2016). At present, chitin degradation and chitinases are playing an important role in a wide variety of biological and biotechnological processes, ranging from the exploitation and environmental clean-up of chitinous wastes to plant defence systems and biological control.

Studies on characterisations and activity of extracellular chitinase from *Trichoderma* species had been started long time ago such as *T. harzianum* (Ulhoa and Peberdy 1991; El-Katatny et al. 2000, 2001; Sandhya et al. 2004), *Trichoderma* sp. (Lima et al. 1999), *T. virens* (Baek et al. 1999). However, studies in *T. asperellum* have only recently begun and only focused on chitinase coding genes, its characterisation and activity (Loc et al. 2011, 2013; Kumar et al. 2012a; Nadarajah et al. 2014; Asad et al. 2015; Wu et al. 2017; Pandian et al. 2018; Liu et al. 2019, etc.).

Many studies have shown *Colletotrichum* is a large genus of *Ascomycete* fungi, containing species that cause serious anthracnose diseases on a wide range of economic value crops such as mango (Rivera-Vargas et al. 2006; Ismail et al. 2015; Mo et al. 2018) and chilli (Than et al. 2008; Oo and Oh 2016; Saxena et al. 2016). *S. rolfsii* fungus was also reported that is the cause of serious stem and pod rot disease in peanut production in many peanut-growing regions (Mehan et al. 1994; Okabe and Matsumoto 2000; Cilliers et al. 2003; Jogi et al. 2016). Meanwhile, peanut, mango and chilli are three of agricultural crops of high economic value in Vietnam. The present work, therefore, purposed to evaluate the antifungal activity of extracellular chitinase from *T. asperellum* PQ34 strain on crop plant (peanut) and fruits (mango and chilli) for potential applications in agriculture and post-harvest storage.

## 2. Materials and methods

### 2.1. Fungal strains and experimental materials

*T. asperellum* PQ34 strain was isolated from the surface soil layer (15–20 cm in depth) under chilli cultivation in Thua Thien Hue province, Vietnam, which exhibited high chitinolytic secretion in the assay of colloidal chitin hydrolytic activity on an agar plate. Species identification for *Trichoderma* was done by PCR amplification of ITS (internal transcribed spacer) region from the fungal genome with ITS1 and ITS4 primers, and then the nucleotide sequence of the amplicon was used to search BLAST in Genbank database (Loc et al. 2011). The strain was stored at −70°C as 40% glycerol stock.

Disease fungal strains (*S. rolfsii* and *Colletotrichum* sp.) were supplied by the Department of Plant Protection, Hue University of Agriculture and Forestry, Hue University, Vietnam.

Healthy mango and chilli fruits, devoid of freckles (0 FPA, freckles per cm^2^ area), and peanut seeds (large and plump seed, bright seed coat, no scratches and over 90% germination rate) were used for research.

Fungicides were not applied at any stage of fruit development.

### 2.2. Fungal culture and chitinase production

Two millilitres of spore suspension (approx. 10^6^ spores/mL) of *T. asperellum* PQ34 was cultured in 100 mL of Czapek-Dox medium supplemented with 10% glucose at 28°C on a shaker with a speed of 180 rpm for 96 h. Mycelia was harvested and rinsed with 2% MgCl_2_, and then with sterile distilled water for three times.

Mycelia was subcultured in Czapek-Dox medium without glucose but supplemented with 1% (*w/v*) colloidal chitin that prepared from native chitin (Sigma-Aldrich) as a carbon source (Shanmugaiah et al. 2008). The culture was incubated at 28°C on a shaker with a speed of 180 rpm. The broth was recovered after 96 h of culture, then filtered with Whatman filter paper (No. 1) and used as a crude enzyme (Harighi et al. 2007). For partial purification, the crude enzyme was precipitated by ammonium sulphate (70% saturation) at 4°C for 2 h, centrifuged at 15,000 rpm at 4°C for 10 min. The pellet was resuspended in 0.1 M sodium acetate (pH 5) and dialysed with MWCO of 12 kDa overnight against 0.05 M sodium acetate (pH 5). Enzyme after partial purification has been used for further studies (Loc et al. 2011).

### 2.3. Chitinase activity assay

Chitinase activity was determined by spectrophotometric method according to Tsujibo et al. (1998) with para-nitrophenyl-β-N-acetyl glucosaminide (pNp-GlcNAc) as a substrate. Seventy microlitres of partially purified enzyme was added to 140 µL of 2.5 mM pNp-GlcNAc that dissolved in 50 mM acetate buffer (pH 6) and incubated at 50°C for 10 min. Hydrolysis was then stopped using 1.4 mL of 0.2 M sodium carbonate. Absorbance was measured at a wavelength of 420 nm. Chitinase activity was defined as the amount of enzyme released 1 µmol *p*-nitrophenol from pNp-GlcNAc during 1 min. *p*-nitrophenol (Sigma-Aldrich) was used as a standard to determine chitinase activity with an estimated regression equation of *y* = 0.673*x* (R^2^ = 0.988), where *y* is *p*-nitrophenol content and *x* is absorbance at 420 nm.

### 2.4. Characterisation of chitinase

The optimal temperature and pH for enzyme activity were investigated in a range of 25–70°C and 4–10, respectively. Buffers for optimal pH determination were 20 mM citrate buffer (pH 4–6), 20 mM phosphate buffer (pH 7–8), and 20 mM glycine-NaOH buffer (pH 9–10). The thermal and pH stability of enzyme were studied by incubating enzyme for 30 min at temperatures of 25–70°C and pH of 4–10 without substrate, enzyme solution was then immediately cooled to 4°C. Effect of metal ions and some surfactants on enzyme activity were tested by incubating enzyme at appropriate temperature and pH for 30 min with 5 mM metal ion (Na^+^, Al^3+^, Fe^2+^, Mg^2+^, Cu^2+^, Co^2+^, Ca^2+^, Zn^2+^, Mn^2+^, or Fe^3+^) or surfactants such as 1% SDS (sodium dodecyl sulphate), 1 mM EDTA (ethylenediaminetetraacetic acid), 1 M urea, 5% DMSO (dimethyl sulphoxide) or 1% Triton X-100. Relative activity of chitinase was determined as described in “chitinase activity assay” section but at optimal temperature and pH. The boiling enzyme solution was used as the blank.

### 2.5. *In vitro* antifungal activity of chitinase

The antifungal activity of chitinase was determined using an assay based upon inhibition of hyphal growth of the phytopathogenic fungi as *S. rolfsii* and *Colletotrichum* sp., all of which have chitin in their cell wall. Petri dish (Φ = 6 cm) containing 1/2 potato dextrose (PD) broth (potato dextrose agar [PDA] without agar) was supplemented with 10–60 U/mL of enzyme and 10 µL of fungal spore suspension (approx. 10^6^ spores/mL). The culture was incubated at 28°C from 36 to 48 h to evaluate the growth of fungus. Mycelium cells were harvested by centrifugation at 4000 rpm for 5 min and rinsed with distilled water to determine fresh biomass, followed by drying at 65°C to a constant weight to determine dry biomass.

### 2.6. Antifungal activity of chitinase in peanut

Healthy peanut (*Arachis hypogaea*) seeds were peeled and treated with 1% sodium hypochlorite for 1 min, then rinsed with sterile distilled water and soaked with enzyme (10–60 U/mL) for 1 h. Finally, the seeds were sown in soil pots that have been inoculated artificially before with *S. rolfsii*, 15 seeds for each treatment, 5 days after sowing the seeds will be germinated. The samples before and after germination were gently taken by hand to observe the appearance of mycelium and count the number of infected samples. The rate of fungal infection (FI) of seeds was evaluated before and after germination.

$$DB(\%)=\frac{NS}{TS}\times 100,$$

where DB: FI before germination, NS: number of fungal-infected seeds, TS: total seeds.

$$DA(\%)=\frac{NP}{TP}\times 100,$$

where DA: FI after germination, NP: number of fungal-infected plants, TS: total plants.

The area under the disease progress curve (AUDPC) was determined by the method of Campbell and Madden (1990).

### 2.7. Antifungal activity of chitinase in mango and chilli fruits

Healthy mango and chilli fruits were collected and rinsed with tap water; they were then treated with 70% ethanol and rinsed again with sterile distilled water.

*Fungal prevention*. Mango and chilli fruits were sprayed with chitinase (10–60 U/mL) to wet, then allow to dry naturally and innoculate artificially with approx. 10^5^ spores of *Colletotrichum* sp./lesion, 10 lesions/fruit (mango) or 1 lesion/fruit (chilli). Fruits were stored in boxes at room temperature (26 ± 2°C). Diameters of the anthracnose lesions (cm) were measured daily to evaluate the level of disease progression.

*Fungal treatment*. Mango and chilli fruits were inoculated artificially before with *Colletotrichum* sp. as described above; after the appearance of the anthracnose lesions on fruits (2–4 days), they will be treated with chitinase from 10 to 60 U/mL. Fruits were stored in boxes at room temperature (26 ± 2°C). Diameters of the anthracnose lesions (cm) were also measured daily to evaluate the level of disease progression.

### 2.8. Statistical analysis

The experiments were conducted with three replications per treatment (n ≥ 30). The data are represented as the mean of the repeats; the means were compared using one-way ANOVA (Duncan’s test at p = 0.05).

## 3. Results

### 3.1. Chitinase production from *T. asperellum* PQ34

**Figure 1.**
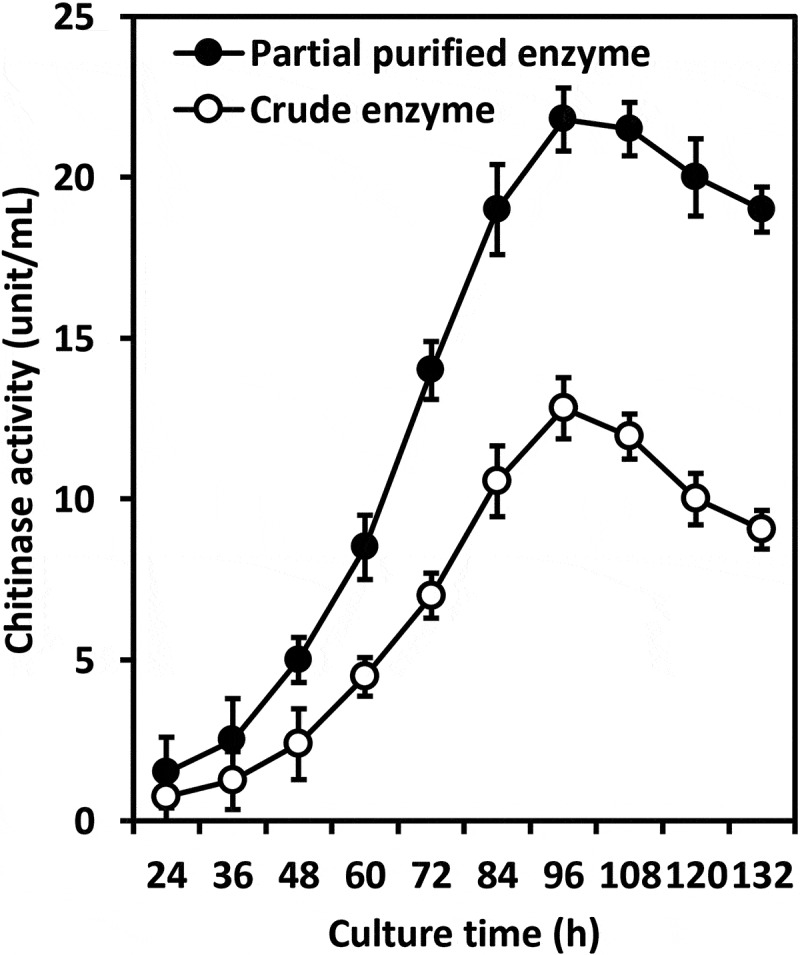
Time course of extracellular chitinase production from *T. asperellum* PQ34.

Data in Figure 1 show a time course of extracellular chitinase production from *T. asperellum* PQ34 during 132 h. Samples were taken periodically for enzyme activity assay. Chitinase activity was present in medium after 24 h of culture at 28°C on a shaker with a speed of 180 rpm and increased continuously with time. The partial purity enzyme reached a maximum total activity of approx. 22 U/mL after 96 h; that is, approx. twofold higher than the crude enzyme.

### 3.2. Characterisations of chitinase

**Figure 2.**
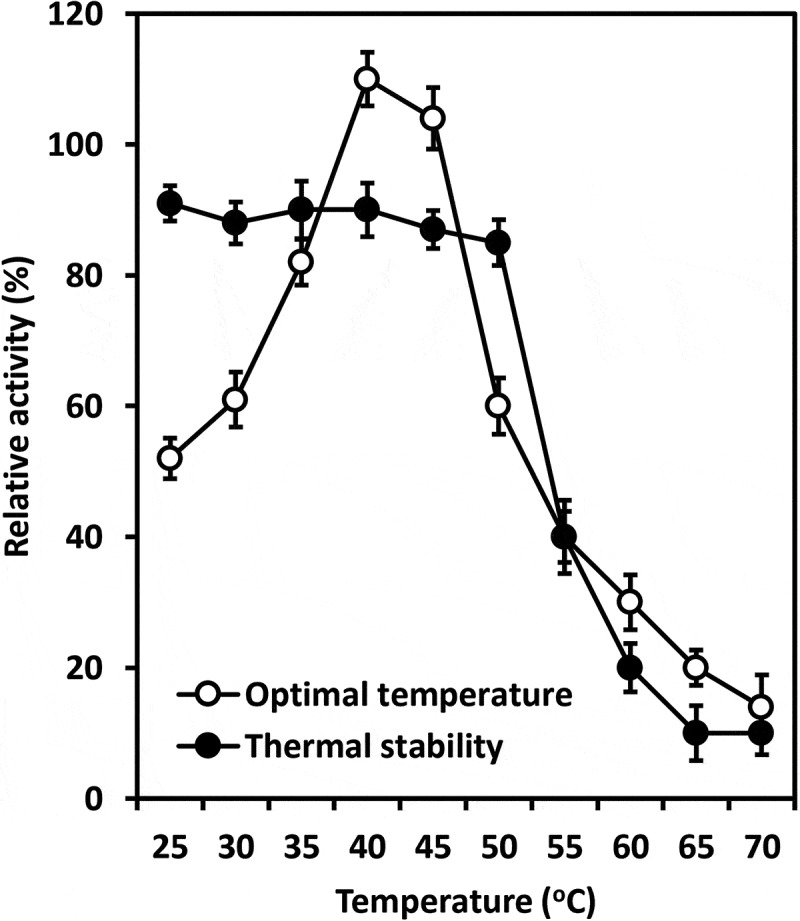
Optimal temperature and thermal stability of chitinase from *T. asperellum* PQ34 at pH 6.

**Figure 3.**
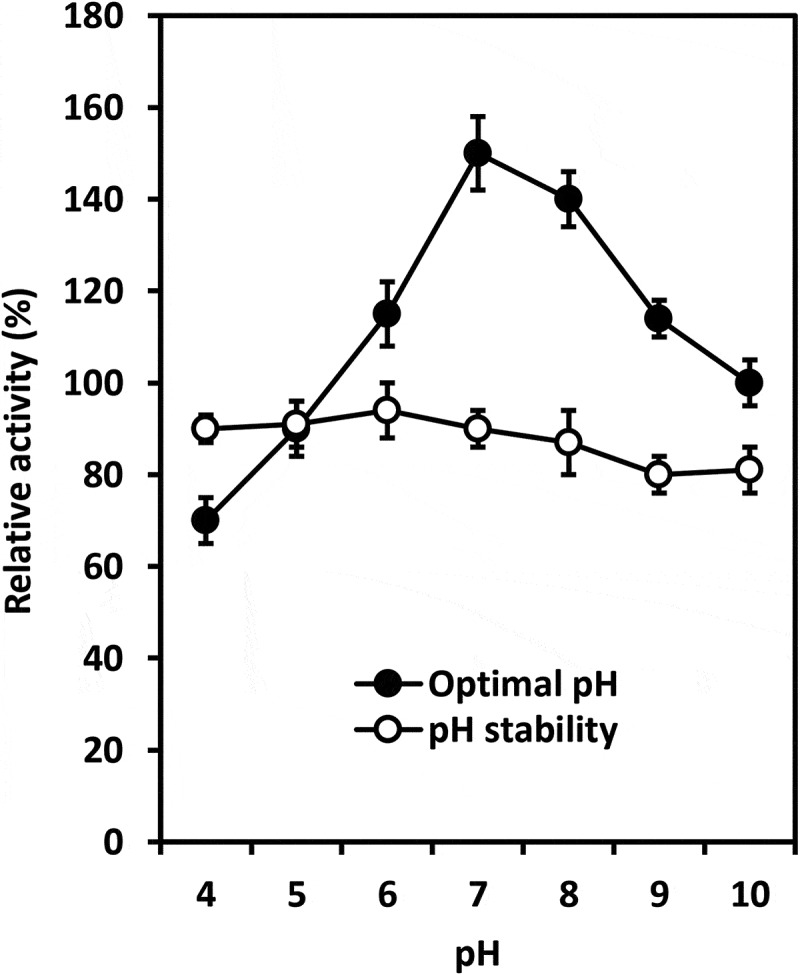
Optimal pH and pH stability of chitinase from *T. asperellum* PQ34 at 40°C.

The study showed that chitinase from *T. asperellum* PQ34 exhibited the optimal temperature and pH of 40°C and 7 with the relative activity of 110% (at 40°C and pH 6) and 150% (at 40°C and pH 7), respectively. The thermal and pH stability of enzyme ranged from 25–50°C and 4–10 with the relative activity of about 90% and from 80% to 90%, respectively (Figures 2 and 3).

**Figure 4.**
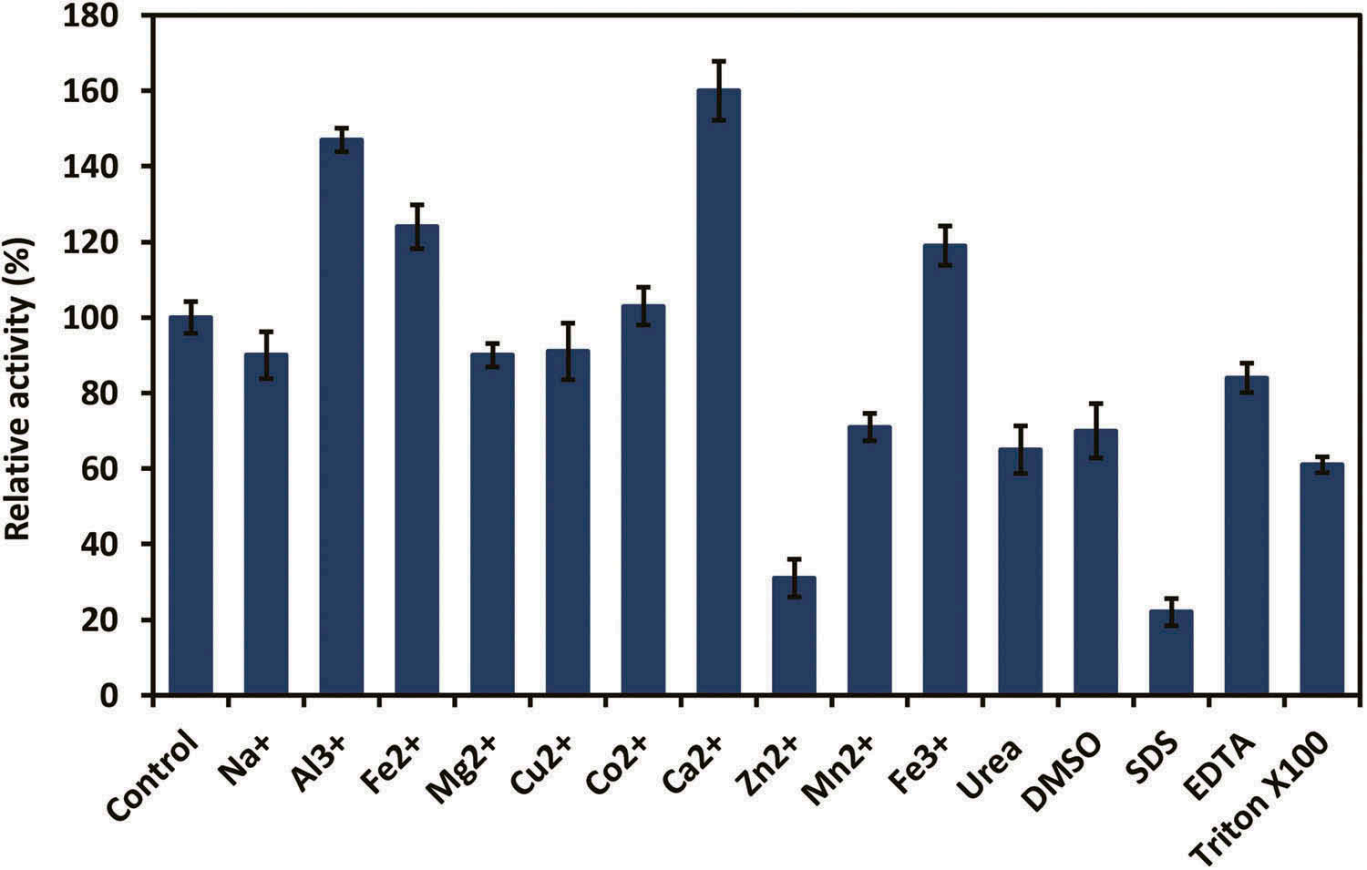
Effect of metal ions and surfactants on chitinase activity from *T. asperellum* PQ34.

As shown in Figure 4, the chitinase activity was significantly reduced by Zn^2+^ and SDS (about 31% and 22%, respectively), whereas it remained about 61–71% under the effect of Triton X-100, urea, DMSO and Mn^2+^. Except for Al^3+^, Fe^3+^, Fe^2+^ and Ca^2+^ that increased clearly chitinase activity (about 119–160%); ions such as Na^+^, Mg^2+^, Cu^2+^ and Co^2+^ had negligible effects on enzyme, relative activity ranged from 90% to 103%.

### 3.3. *In vitro* antifungal activity of chitinase

**Figure 5.**
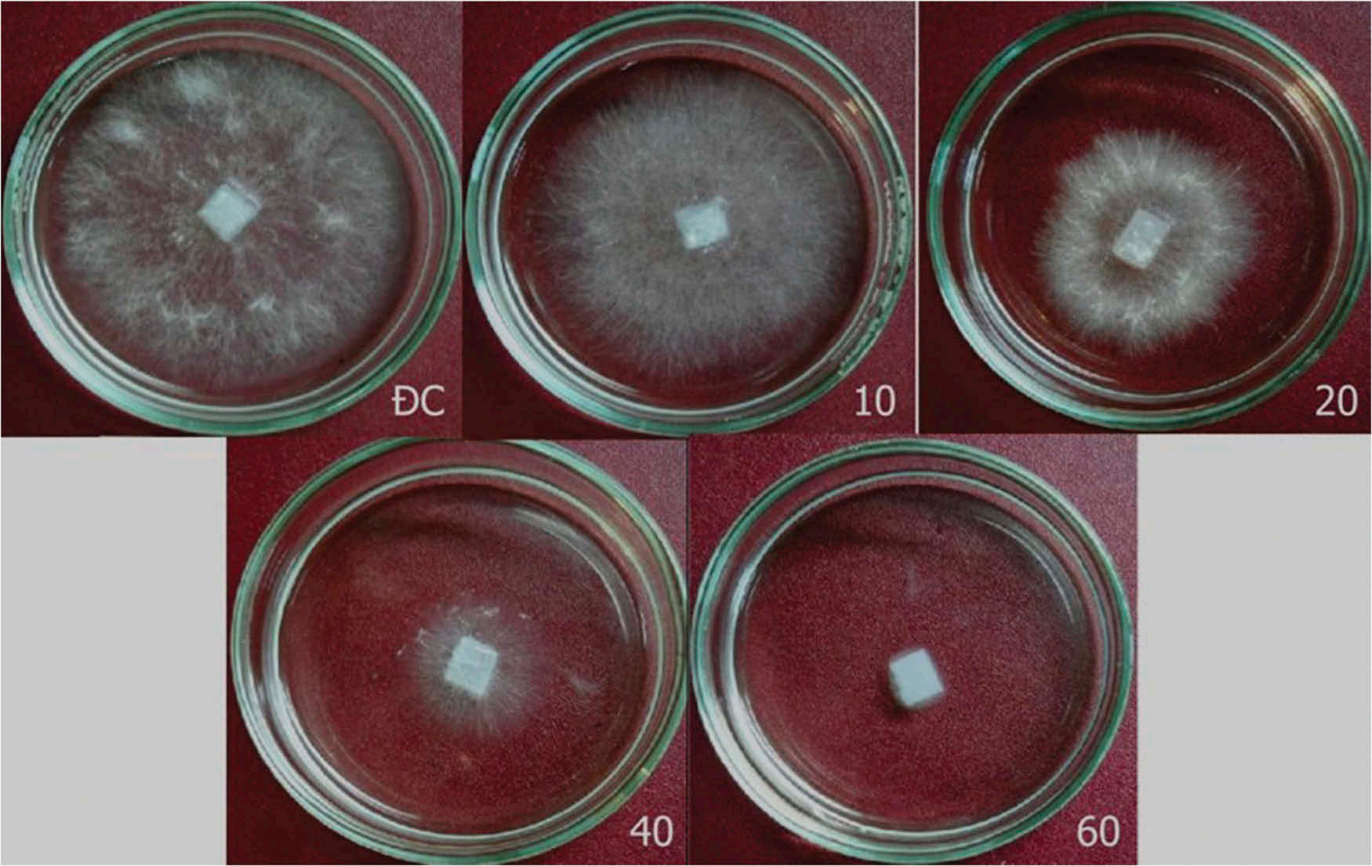
Effect of chitinase from *T. asperellum* PQ34 on *in vitro* growth of *S. rolfsii*. ĐC: control without chitinase, 10–60: chitinase from 10 to 60 U/mL.

Results in Table 1 show that chitinase strongly inhibited the growth of *Colletotrichum* sp. and *S. rolfsii*. Fresh biomass of *Colletotrichum* sp. and *S. rolfsii* significantly reduced approximately 89% (~0.08 g vs 0.72 g) and 27% (~0.9 g vs 1.2 g) for 10 U/mL chitinase treatment, respectively. When treated with 60 U/mL chitinase, growths of *Colletotrichum* sp. and *S. rolfsii* were drastically inhibited, approximately 95% (~0.4 g vs 0.72 g) and 97% (~0.4 g vs 1.2 g), respectively. Figure 5 illustrates the effect of chitinase from 10 to 60 U/mL on the growth of *S. rolfsii*.

**Table 1.** *In vitro* antifungal activity of chitinase from *T. asperellum* PQ34.

Chitinase (U/mL)	*Colletotrichum* sp.		*S. rolfsii*	
	FB (g)	DB (g)	FB (g)	DB (g)
10	0.080^b^	0.008^b^	0.873^ab^	0.007^ab^
20	0.067^b^	0.006^b^	0.504^bc^	0.005^b^
40	0.049^b^	0.005^b^	0.272^cd^	0.003^c^
60	0.037^b^	0.003^b^	0.039^d^	0.001^d^
Control	0.720^a^	0.015^a^	1.201^a^	0.009^a^

Different letters in a column show significant statistical differences with Duncan’s test (*p* < 0.05). This note is also used for all tables. FB: fresh biomass, DB: dry biomass.

### 3.4. Antifungal activity of chitinase in peanut

**Figure 6.**
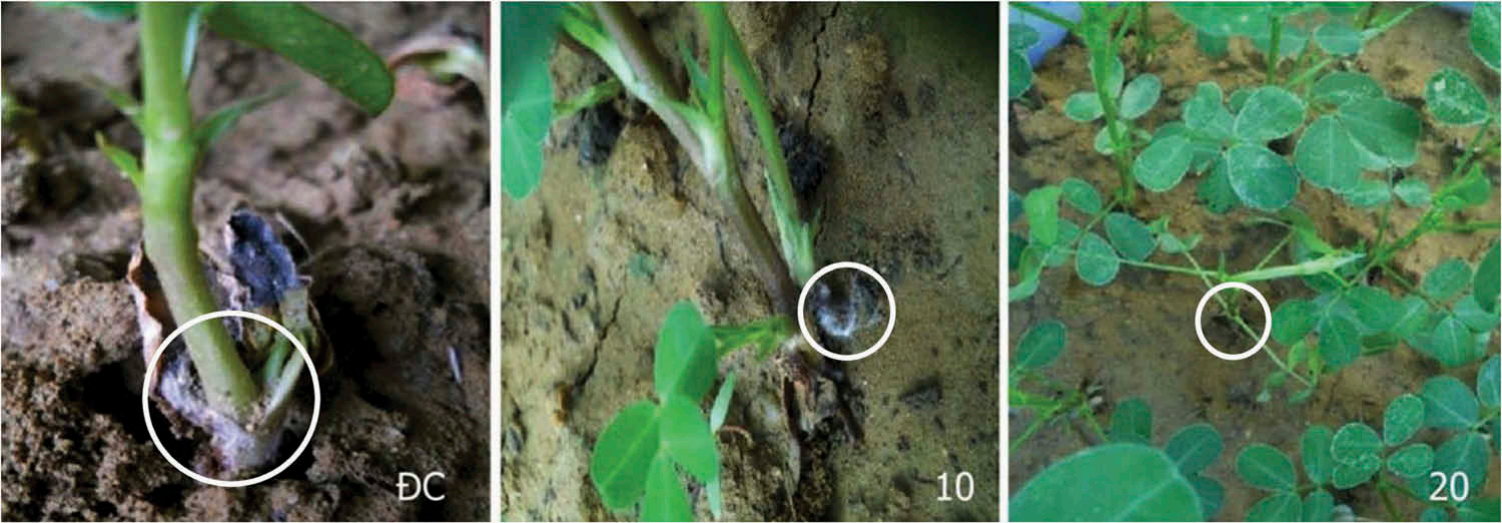
Effect of chitinase from *T. asperellum* PQ34 on the growth of *S. rolfsii* in peanuts. ĐC: control without chitinase, 10 and 20: chitinase of 10 and 20 U/mL, respectively.

*T. asperellum* PQ34 chitinase significantly inhibited the growth of *S. rolfsii* in peanuts. Pre-treated seeds with chitinase (10–60 U/mL) had a very low rate of fungal infection compared to control, before and 30 days after germination, respectively, 2.22–8.89% and 2.22–7.32% (control: 13.33–17.95%). Especially, chitinase from 20 to 60 U/mL drastically reduced the fungal-infected rate in plants (2.22–4.6%) (Figure 6). The AUDPC also reached the lowest in chitinase treatments from 20 to 60 U/mL compared to control, 16.67–34.52 vs 185.9. The antifungal activity of chitinase from 20 to 60 U/mL was not significantly different with *p*> 0.05 (Table 2).

**Table 2.** Antifungal activity of chitinase against *S. rolfsii* in peanuts.

Chitinase (U/mL)	Percentage of fungal-infected plants			
	Before germination	After germination		
		15 days	30 days	AUDPC
10	8.89^a^	4.76^ab^	7.32^ab^	102.56^ab^
20	2.22^b^	0.00^b^	2.38^b^	17.86^b^
40	2.22^b^	0.00^b^	2.22^b^	16.67^b^
60	2.22^b^	0.00^b^	4.60^b^	34.52^b^
Control	13.33^a^	5.13^a^	17.95^a^	185.90^a^

### 3.5. Anthracnose prevention on mango and chilli fruit

**Figure 7.**
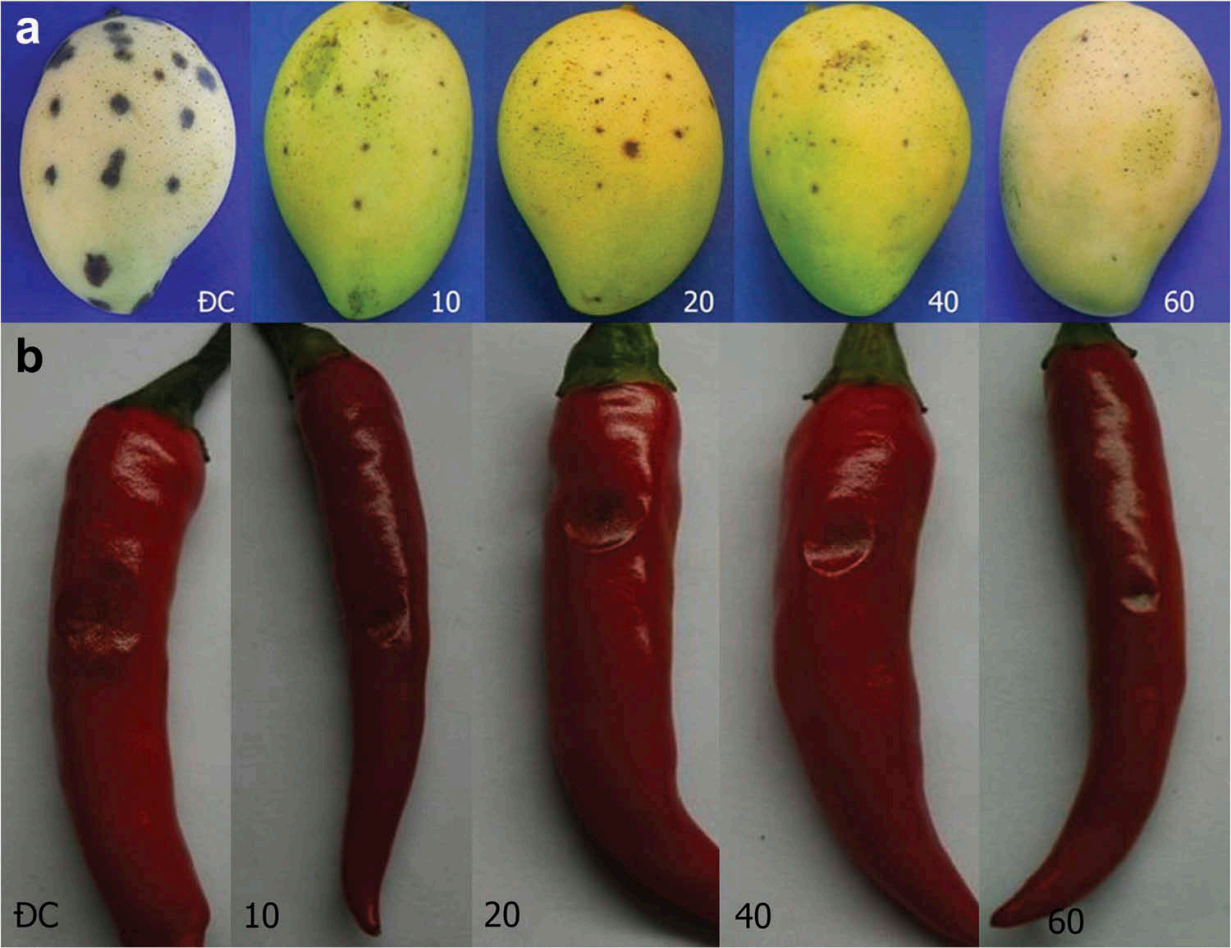
Lesions on mango (a) and chilli (b) fruits after 96 h of *Colletotrichum* sp. infection. ĐC: control without chitinase. 10–60: fruits were pre-treated with chitinase from 10 to 60 U/mL.

Overall, *T. asperellum* PQ34 chitinase is capable of preventing the development of anthracnose on both mango and chilli. After 96 h of chitinase treatment, there were signs of fungal infection but at different levels on mango fruits (Figure 7(a)). In the control group, the lesion appeared after 48 h of infection, whereas lesions only began to appear in enzyme treatments after 96 h of inoculation. The level of disease progression in treated groups was also slower than the control; the lowest AUDPC index belongs to the treatment of 40 U/mL chitinase (Table 3). These results showed that chitinase could prevent anthracnose that caused by *Colletotrichum* sp. on mango up to 72 h after treatment.

**Table 3.** Inhibition effect of chitinase to *Colletotrichum* sp. on mango fruit.

	Diameter of brown spot (cm)									
Chitinase (U/mL)	0 h	24 h	48 h	72 h	96 h	120 h	144 h	168 h	192 h	AUDPC
10	-	-	-	0.1	0.2	0.32	0.41	0.69	0.84	46.56^b^
20	-	-	-	-	0.12	0.22	0.34	0.54	0.60	35.16^c^
40	-	-	-	-	0.11	0.19	0.27	0.36	0.46	26.52^d^
60	-	-	-	-	0.10	0.16	0.29	0.49	0.70	31.16^cd^
Control	-	-	0.1	0.15	0.35	0.47	0.56	0.76	0.91	58.80^a^

The chitinase treatments for chilli also obtained similar results in mango; the effective of fungal prevention was in about 72 h. The lesions appeared in the control group at 72 h, and after 144 h, they spread, stuck together and rotted. Treatment of chitinase from 10 to 60 U/mL was only effective before 96 h (Figure 7(b) and Table 4). The lowest AUDPC also belongs to the 40 U/mL treatment similar to mango.

**Table 4.** Inhibition effect of chitinase to *Colletotrichum* sp. on chilli fruit.

	Diameter of rotten spot (cm)									
Chitinase (U/mL)	0 h	24 h	48 h	72 h	96 h	120 h	144 h	168 h	192 h	AUDPC
10	-	-	-	-	0.2	0.35	0.65	1.05	2.05	76.20^ab^
20	-	-	-	-	0.15	0.30	0.50	0.80	1.90	63.00^bc^
40	-	-	-	-	0.1	0.20	0.30	0.80	1.55	51.00^c^
60	-	-	-	-	0.1	0.15	0.40	0.80	1.55	52.20^c^
Control	-	-	-	0.1	0.17	0.50	0.95	1.35	2.25	96.30^a^

### 3.6. Anthracnose treatment in mango and chilli fruit

**Figure 8.**
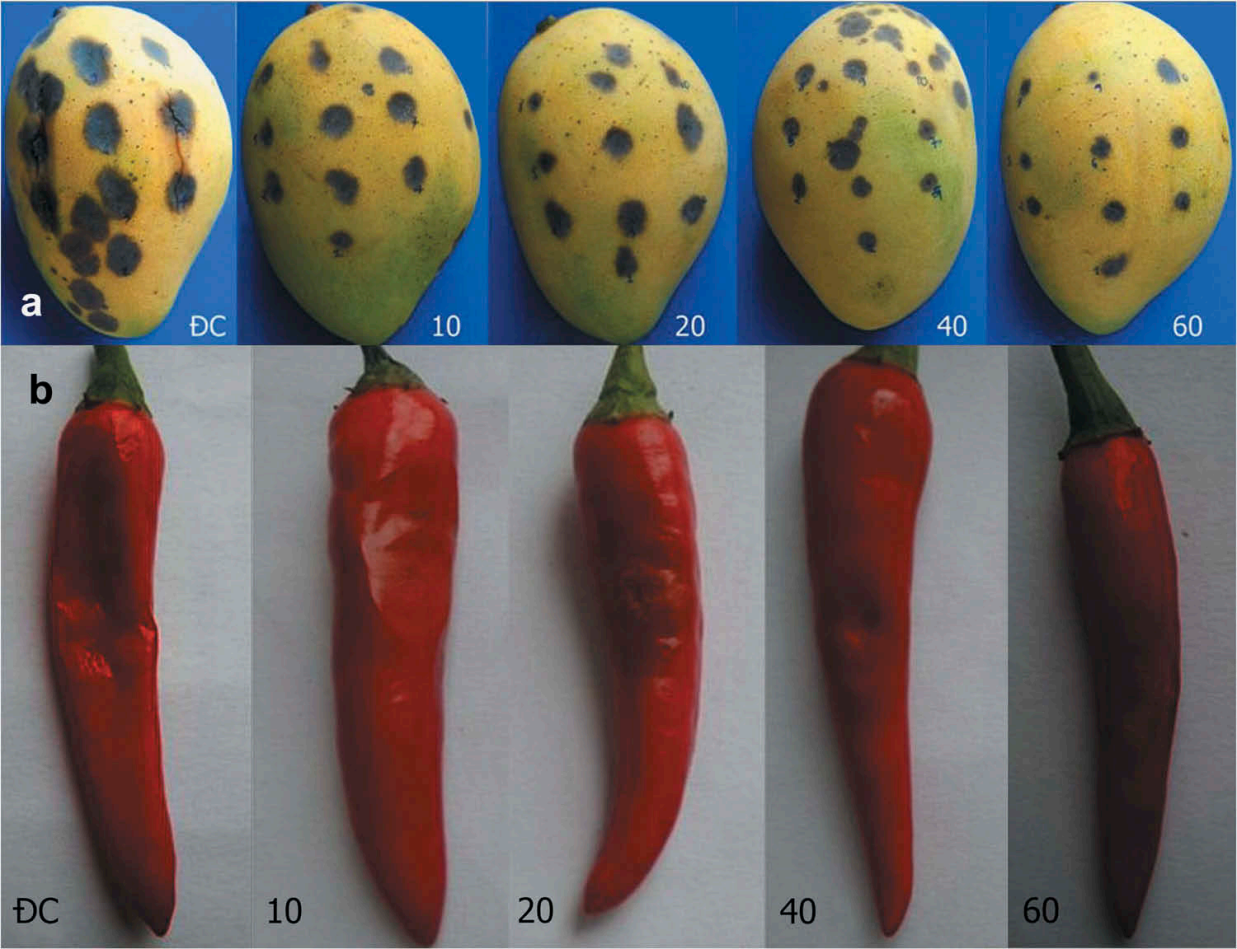
Treatment of anthracnose for mango (a) and chilli (b) fruits after 168 h. ĐC: control with chitinase. 10–60: fruits were treated with chitinase from 10 to 60 U/mL.

The higher activity of chitinase significantly inhibited the growth of fungus on mango. After 96 h of treatment with chitinase from 40 to 60 U/mL, the diameters of lesions on mango fruits were only in the range of 0.88–0.90 cm (control: 1.67 cm). The progression of AUPDC was only half that of the control, 41.16 vs 81.84 (Figure 8(a) and Table 5).

**Table 5.** The effect of chitinase on the progression of lesions on mango.

	Diameters of lesions (cm)					
		After treatment				
Chitinase (U/mL)	Before treatment	24 h	48 h	72 h	96 h	AUDPC
10	0.11	0.23	0.50	0.84	1.24	53.88^b^
20	0.10	0.28	0.47	0.78	1.13	51.48^b^
40	0.11	0.22	0.38	0.62	0.88	41.16^c^
60	0.10	0.16	0.37	0.59	0.90	38.88^c^
Control	0.18	0.46	0.81	1.21	1.67	81.84^a^

Similar to mango fruit, the development of fungal pathogens on chilli was partially inhibited by chitinase. However, the growth rate of disease fungi on chilli fruit was faster, and the diameters of the lesions after 96 h of chitinase treatments from 40 to 60 U/mL were 1.35–1.45 cm (control: 2.85 cm). AUPDC of 40–60 U/mL chitinase treatment was only half that of the control, 66.6–70.2 vs 143.4 (Figure 8(b) and Table 6).

**Table 6.** The effect of chitinase on the progression of lesions on chilli.

	Diameters of lesions (cm)					
		After treatment				
Chitinase (U/mL)	Before treatment	24 h	48 h	72 h	96 h	AUDPC
10	0.15	0.50	0.95	1.50	1.80	95.40^b^
20	0.13	0.60	0.90	1.35	1.65	91.20^bc^
40	0.10	0.35	0.65	1.00	1.45	66.60^c^
60	0.10	0.40	0.75	1.05	1.35	70.20^bc^
Control	0.12	0.80	1.35	2.25	2.85	143.40^a^

## 4. Discussion

Sandhya et al. (2004) fermented *T. harzianum* TUBF 966 strain at 30°C for 96 h and obtained extracellular chitinase with a maximum activity of 14.7 U/mL. Asad et al (2105) found a peak of chitinolytic activity (173 U/mL) by *T. asperellum* after 96 h of culture at 25°C which was the highest among all the isolates, whereas that of *T. harzianum* was 117 U/mL after 72 h of culture at the same temperature. In which, one unit of the activity was defined as the amount of enzyme that to produce 1 mmol of the product per mg of the protein per hour. A *T. harzianum* strain from the study of Urbina-Salazar et al. (2018) also gave the chitinase yield of 261.5 mU/L per day. Similar to the *T. asperellum* strain in the study of Asad et al. (2015), *T. asperellum* PQ34 also required an incubation period of 96 h but at a higher temperature of 28°C to produce the chitinase with a maximum activity (about 22 U/mL). The suitable incubation period or temperature of mentioned *T. harzianum* strains are different from *T. asperellum* PQ34.

In general, the characteristics of chitinases from *Trichoderma* species are relatively different. Chitinase from *T. asperellum* PQ34 strain had the optimal temperature and pH are 40°C and 7, the thermal and pH stability ranged from 25–50°C and 4–10, respectively. Meanwhile, chitinase from *T. harzianum* Rifai (T24) strain was stable at 30°C and it is rapidly inactivated at 60°C (El-Katatny et al. 2001). The optimal temperature and pH for chitinolytic activity of *T. viride* were 50°C and 5, respectively. The chitinase produced by this fungal strain was stable at temperatures of 40–50°C and pH 6–7 (Ekundayo et al. 2016). According to Ulhoa and Peberdy (1992), of the metal ions tested, only Zn^2+^ had a significant inhibitory effect on chitinase from *T. hazianum* 39.1 strain, and its relative activity was about 43%. No significant loss of enzyme activity was observed after incubation with the EDTA and other metal ions as Ca^2+^, Cu^2+^, Fe^2+^, K^+^, Mg^2+^, Mn^2+^ and Na^+^; the relative activity ranged from 78% to 99%. Ekundayo et al. (2016) found the relative activity of chitinase from *T. viride* was reduced when EDTA and MnCl_2_ were used. This enzyme was most sensitive to EDTA followed by MnCl_2_ and it achieved the maximum activity when CaCl_2_ was used. The effects of metal ions and surfactants on the chitinase activity from *T. asperellum* PQ34 strain were relatively different to chitinase from various *Trichoderma* species. Chitinase activity from PQ34 strain was significantly reduced by Zn^2+^ and SDS, whereas it remained about 61–71% under the effect of Triton X-100, urea, DMSO and Mn^2+^. Except for Al^3+^, Fe^3+^, Fe^2+^ and Ca^2+^ that increased clearly chitinase activity; ions such as Na^+^, Mg^2+^, Cu^2+^ and Co^2+^ had negligible effects on enzyme activity.

Lima et al. (1999) showed the endochitinase (CHIT 46) from *Trichoderma* sp. drastically affected the cell walls of *S. rolfsii* and *Rhizoctonia solani*. According to El-Katatny et al. (2000), chitinase of *T. harzianum* has significantly inhibited the growth of *S. rolfsii* (up to 61.8%). Kumar et al. (2012b) also found the antagonistic activity of chitinase from *Trichoderma* spp. against root rot and foliar pathogens. Pacheco et al. (2016) evaluated the efficacy of six *Trichoderma* isolates to control sclerotium wilt (*S. rolfsii*) of common bean (*Phaseolus vulgaris*) and found that three of them (*T. hazianum, T. longibrachiatum* and *T. reesei*) were more efficient in reducing sclerotial germination. Marques et al. (2018) isolated two strains CEN1245 and CEN1274, both belonging to the species *T. brevicompactum*, exhibiting a broad spectrum against *S. rolfsii, C. gloesporioides*, and the other disease fungi. De la Cruz-quiroz et al. (2018) studied *in vitro* antagonistic activity in dual culture detected that *T. asperellum* (T2-32) strain displayed the highest capacity of inhibition against *Colletotrichum* gloeosporioides up to 22.5%. However, it had no effect on the spores of *Colletotricum* isolates. Our study showed that chitinase at 60 U/mL inhibited nearly completely *in vitro* growth of *Colletotrichum* sp. and *Sclerotium rolfsii*. In peanut plants, 20 U/mL of chitinase significantly reduced the incidence of *S. rolfsii* infection. The fungal infection incidence of seedsbefore germination and 30 days after germination was only 2.22% and 2.38%, while the control was 13.33% and 17.95%. Besides, this enzyme can also prevent anthracnose that is caused by *Colletotrichum* sp. on both mango and chilli fruits up to 72 h after enzyme pre-treatment at 40 U/mL. In mango and chilli fruits infected with anthracnose, 40 U/mL dose of chitinase inhibited growth of fungi after 96 h of treatment.

In addition to enzymes such as chitinase, glucanase and protease, some reports showed that *Trichoderma* species also secreted trichodermin, an antibiotic that has antifungal activity, into medium during fermentation, such as *T. reesei* (Watts et al. 1988), *T. brevicompactum* (Shentu et al. 2014), *T. koningiopsis* (Leylaie and Zafari 2018). In this work, we have not investigated the production of trichodermin in *T. asperellum* PQ34. Chitinase was partially purified by ammonium sulphate precipitation which can still contain trichodermin; therefore, antifungal activity may not be only of the enzyme.

In conclusion, the characteristics and antifungal activity of chitinase from *Trichoderma* species are relatively different. Chitinase of *T. asperellum* PQ34 can inhibit the growth of two strains of *S. rolfsii* and *Colletotrichum* sp. has opened up the potential for environmental friendly fungicides later on.
